# A novel method for biopolymer surface nanostructuring by platinum deposition and subsequent thermal annealing

**DOI:** 10.1186/1556-276X-7-671

**Published:** 2012-12-12

**Authors:** Petr Slepička, Petr Juřík, Zdeňka Kolská, Petr Malinský, Anna Macková, Iva Michaljaničová, Václav Švorčík

**Affiliations:** 1 Department of Solid State Engineering, Institute of Chemical Technology, Prague, 166 28, Czech Republic; 2 Faculty of Science, J.E. Purkyně University, Ústí nad Labem, 400 96, Czech Republic; 3 Nuclear Physics Institute, Academy of Sciences of the Czech Republic, Řež, 250 68, Czech Republic

**Keywords:** Nanopatterning, Surface morphology, Biopolymer, Platinum sputtering, Thermal annealing

## Abstract

A novel procedure for biopolymer surface nanostructuring with defined surface roughness and pattern dimension is presented. The surface properties of sputtered platinum layers on the biocompatible polymer poly(l-lactic acid) (PLLA) are presented. The influence of thermal treatment on surface morphology and electrical resistance and Pt distribution in *ca.* 100 nm of altered surface is described. The thickness, roughness and morphology of Pt structures were determined by atomic force microscopy. Surface sheet resistance was studied by a two-point technique. It was the sequence of Pt layer sputtering followed by thermal treatment that dramatically changed the structure of the PLLA’s surface. Depending on the Pt thickness, the ripple-like and worm-like patterns appeared on the surface for thinner and thicker Pt layers, respectively. Electrokinetic analysis confirmed the Pt coverage of PLLA and the slightly different behaviour of non-annealed and annealed surfaces. The amount and distribution of platinum on the PLLA is significantly altered by thermal annealing.

## Background

Polymer-metal composites [1] are becoming an attractive subject because of their unique surface morphology and electric properties. They can be made on the base of polymeric films metalized from one or both sides with a noble metal (gold or platinum) [2,3]. These structures can be applied in many different parts of advanced electromechanical application, such as biomimetic robots [4] or actuators. Metal nanolayers on polymers were used in applications for LCD technology [5]. Metal-polymer composites were studied as a key material for transistor construction in electronics industry [6]. The increasing interest in the field of nanomaterials has created novel or advanced analytical techniques capable of characterizing the materials in the nanostructure scale and preparing functionalized nanostructured materials [7]. Methods for metal nanolayer preparation can be divided into several branches, involving, e.g. sputtering [8,9], evaporation [10] or electrochemical methods. It has been reported frequently that platinum thin film patterns, like temperature sensors or heaters, degrade at temperatures in the range of 500°C to 900°C, which can lead to functional failure of the microdevices on which these patterns are deposited [11]. Polymer-metal structures are of great importance in thermal management of microsystems [11]. The biopolymer poly(l-lactic acid) (PLLA) in combination with various types of treatment procedures, grafting or metal layer deposition, can be applied in the preparation of substrates for tissue engineering [12,13]. Different polymer substrates (e.g. PDMS) can, on the contrary, play a significant role in metal superlattice preparation [14]. Thermal treatment of metal nanostructures such as Pt and Au may also lead to bimetallic nanostructures [15]. The potential applications of polymer-metal nanocomposites and nanoparticles can be found in electronics or biomedical engineering [16-22].

In this paper, we present a simple and cheap method for biopolymer surface nanostructuring by platinum nanostructure deposition and subsequent thermal annealing of the biopolymer surface. The electrical properties, zeta potential, chemical structure and surface morphology of ripple composites are introduced.

## Methods

The biopolymer PLLA (density 1.25 g cm^−3^, glass transition temperature (*T*_g_) = 60°C, crystallinity 60% to 70%, 50-μm-thick foils, supplied by Goodfellow, Ltd., Huntingdon, UK) was used for the present experiments. The platinum layers on the PLLA substrate were deposited from a Pt target (99.999%) by means of diode sputtering technique (BAL-TEC SCD 050 equipment, BalTec Maschinenbau AG, Pfäffikon, Switzerland). The theoretical sputtering rate of the SCD 050 for platinum is 0.15 nm s^−1^. Typical sputtering conditions were as follows: room temperature, time 5 to 500 s, total argon pressure of about 5 Pa, electrode distance of 50 mm and current of 20 mA. For the measurement of the Pt layer thickness, the metal was deposited under the same conditions on a glass substrate and measured with an atomic force microscope (AFM). Typically, five measurements on three scratches each were accomplished on each sample.

The electrical discontinuity/continuity of the as-sputtered and as-sputtered + heated (at 60°C) platinum layers was examined by measuring electrical sheet resistance (*R*_s_). For determination of *R*_s_ by standard Ohm’s method, a KEITHLEY 487 pico-ammeter (Cleveland, OH, USA) was used [9]. Two Au contacts (about 50 nm thick) were sputtered on the layer’s surface for resistance measurement. Typical error of the measurement was ±5%. Thermal treatment of the polymers was accomplished in a BINDER thermostat (Tuttlingen, Germany). The samples were heated for 60 min at 60°C, and then they were cooled down to room temperature.

The surface morphology was examined using an AFM. The AFM images were taken under ambient conditions on a Bruker Corporation CP-II setup (Santa Barbara, CA, USA) [12]. *R*_a_ represents the arithmetic average of the deviations from the centre plane of the sample. Four areas of each sample were scanned in order to obtain representative data.

Rutherford back scattering (RBS) analyses were performed on a Tandetron 4130MC accelerator using 1.7-MeV ^4^He ions (High Voltage Engineering Europa, Amersfoort, The Netherlands). The measurements were performed in IBM geometry with an incident angle of 0° and a laboratory scattering angle of 170°. The typical energy resolution of the spectrometer was FWHM = 15 keV. The RBS spectra were evaluated using SIMNRA and GISA software [13]. The RBS measurement was realized at the CANAM infrastructure.

The zeta potential of the samples was determined using the SurPASS Instrument (Anton Paar, Graz, Austria). The samples were studied inside an adjustable gap cell with an electrolyte (0.001 mol dm^−3^ KCl) at a temperature of 25°C and pH = 6.0. For each measurement, a pair of polymer foils with the same top layer was fixed on two sample holders (with a cross section of 20 × 10 mm^2^ and a gap of 100 μm). All samples were measured four times with a relative error of ±10%. For zeta potential determination, we applied two methods (streaming potential and streaming current) and two equations for zeta potential calculation (Helmholtz-Smoluchowski and Fairbrother-Mastins) [23].

## Results and discussion

**Figure 1:**
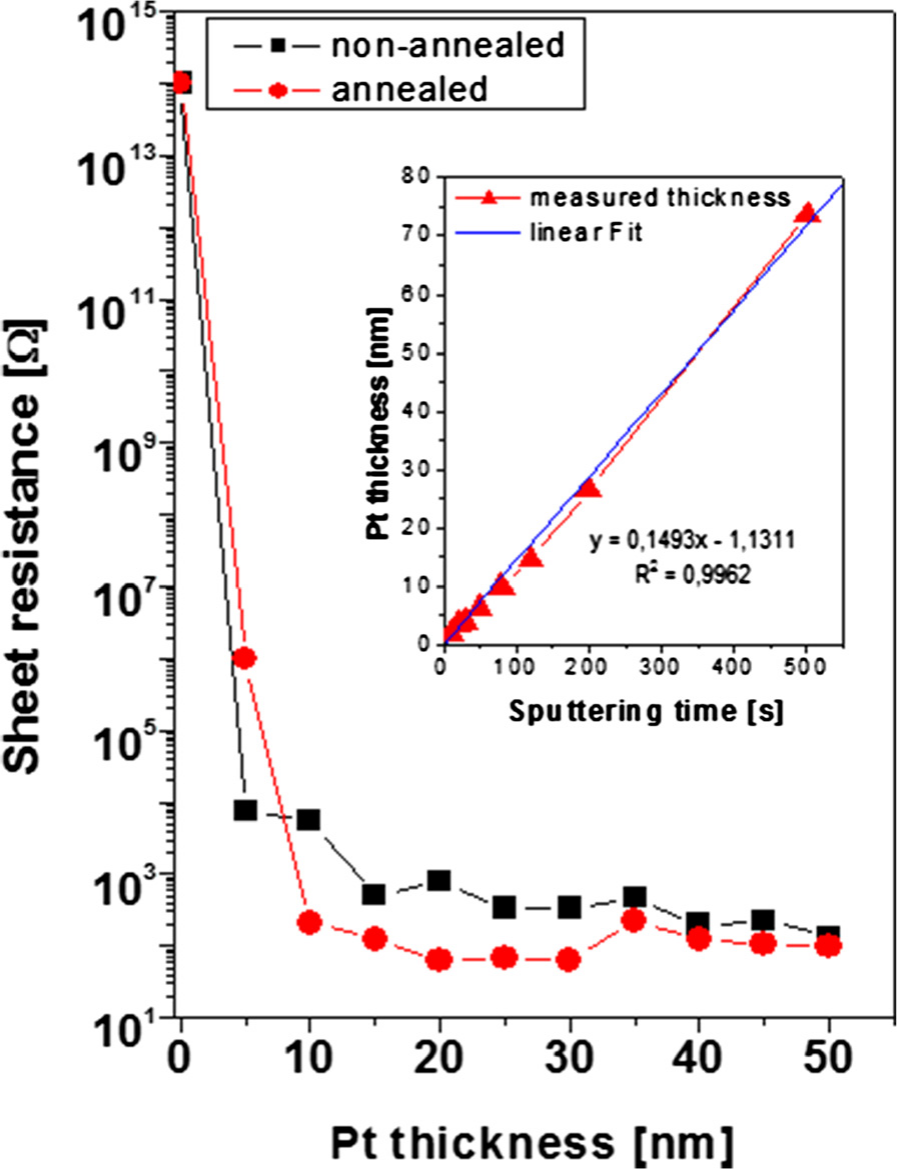
**Dependence of *R ***_**s**_**on thickness of Pt sputtered layer for non-annealed and annealed samples.** The embedded graph shows the dependence of layer thickness on Pt sputtering time, the dependence being fitted with standard linear model.

The transition to an electrically continuous layer was determined for the Pt layer deposited on pristine PLLA and the same set of samples heated at 60°C (Figure 1). The transition of the electrical continuity of the sputtered Pt layer is connected with the saturation of sheet resistance.

As it is apparent from Figure 1, thermal annealing leads to the shift of the point of electrical continuity. The Pt becomes electrically continuous in the thickness above 5 nm, while in the PLLA/Pt heated at 60°C, the point of electrical continuity has shifted to the Pt thickness of 10 nm. The Pt deposited on PLLA and heated exhibits in the area of electrical continuity lower values than the layer on the non-heated sample; for higher thicknesses, the convergence is apparent. The changes of electrical continuity are probably caused by the formation of the PLLA/Pt composite, which will be discussed further (RBS analysis), and by changes in surface morphology. The errors of measurement are introduced, but they are within the graph points (did not exceed 3%). The dependence of Pt thickness on sputtering time was proved to be linear (see embedded graph in Figure 1). The dependence was fitted with standard mathematical regression with a confidence interval better than 0.99.

**Figure 2:**
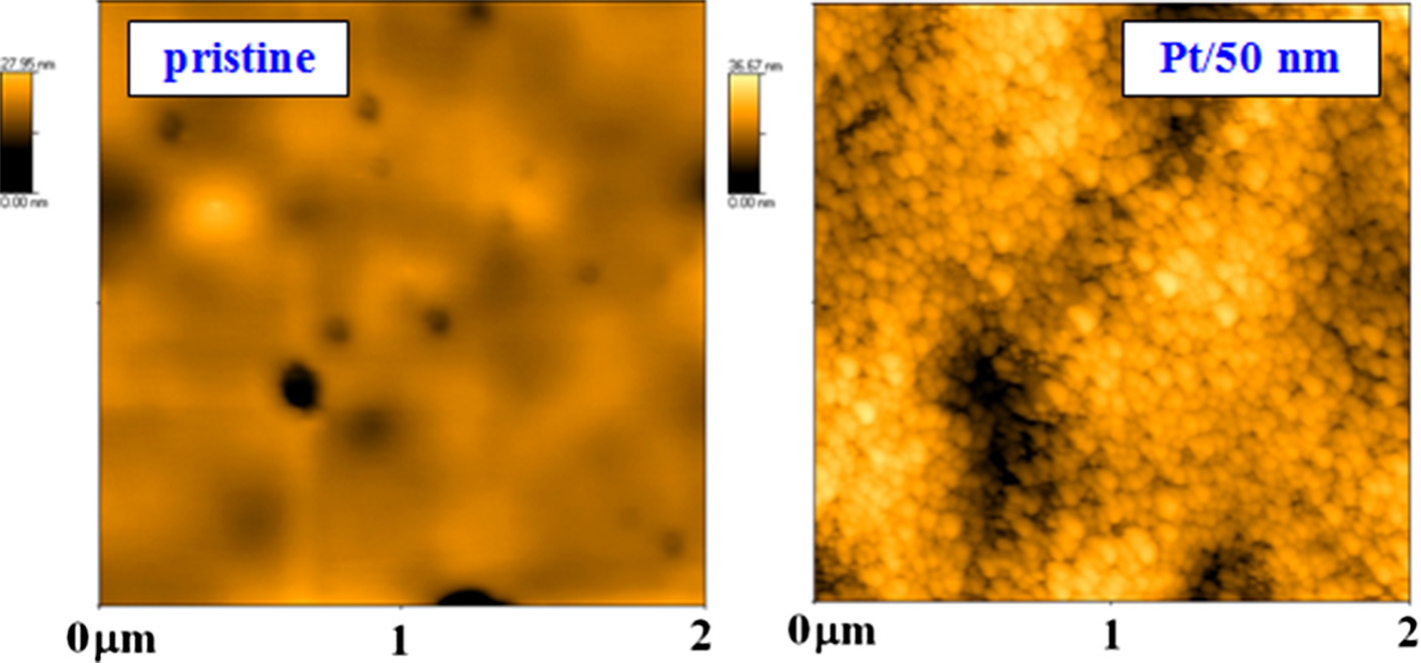
**The surface morphology (2 × 2 μm**^**2**^**detail) of PLLA samples determined by AFM.** The detail of pristine PLLA sample (left) and PLLA deposited with Pt (thicknesses 50 nm, right) is introduced.

Surface morphology of Pt/PLLA samples was studied with AFM. The surface morphology of pristine PLLA and the Pt/PLLA sample with 50 nm of Pt film is, for illustration, introduced in Figure 2. From this figure, the dramatic change in surface morphology after Pt deposition is apparent. Sputtered Pt structures grow in a form of globular structures. In comparison to other methods (e.g. surface grafting), the method of PLLA-Pt sputtering combined with annealing exhibits the advantage of the PLLA-metal composite preparation, which can be easily manufactured and modified.

**Figure 3:**
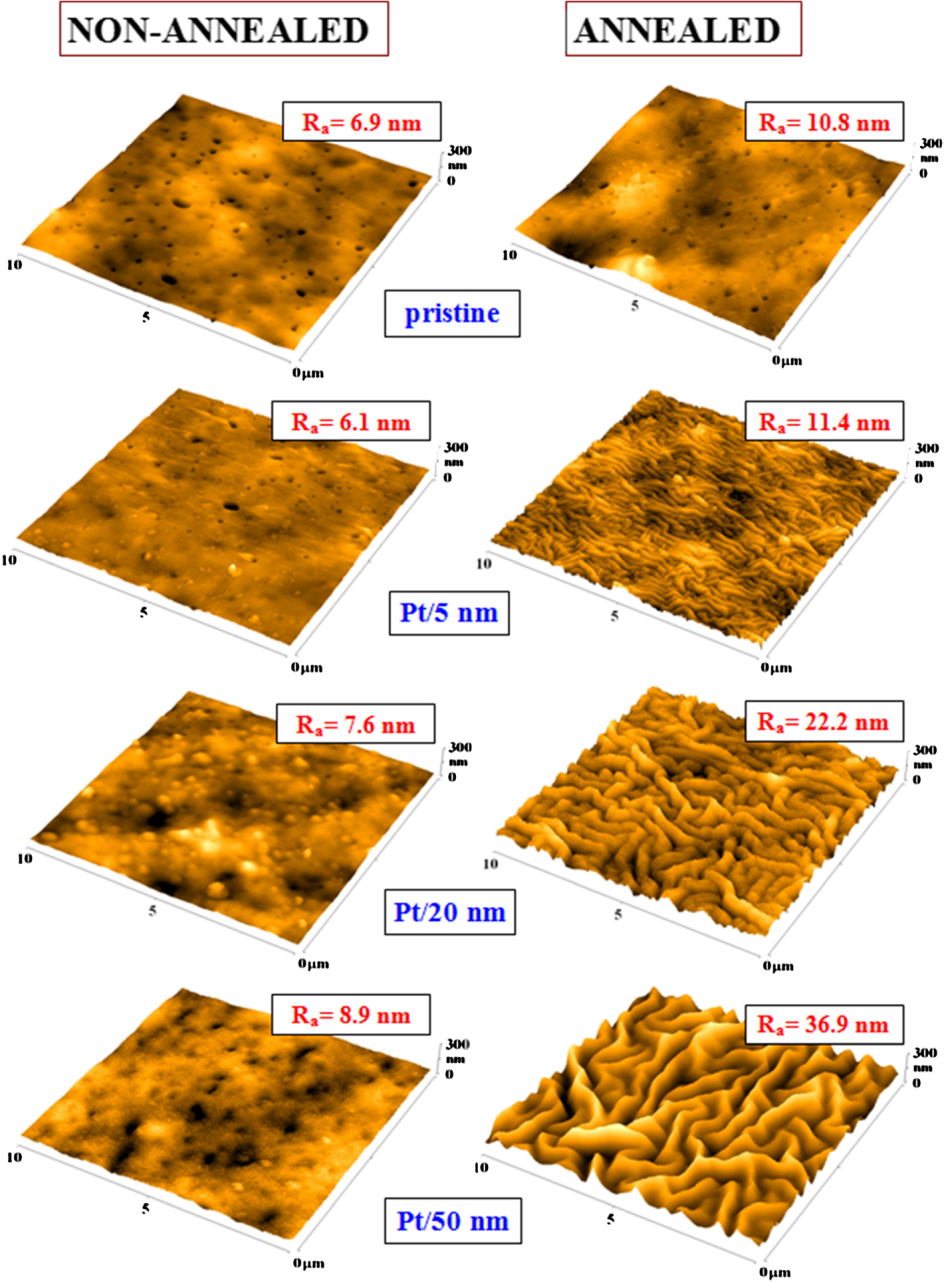
**The surface morphology of non-annealed (left) and annealed (right) PLLA samples determined by AFM.** The PLLA samples were deposited with Pt with thicknesses 5, 20 and 50 nm and then heated at 60°C. The thermally annealed pristine PLLA is also shown for comparison. *R*_a_ represents the arithmetic surface roughness.

The change of surface morphology of pristine PLLA and PLLA sputtered with platinum (thickness 5, 20 and 50 nm) non-annealed and thermally annealed samples is introduced in Figure 3. The deposited Pt strongly influences the surface structure of PLLA when heated. Annealing of pristine PLLA does not have any significant influence on the surface morphology: the surface roughness is mildly increased. Thermal annealing of the sputtered polymer surface results to Pt nanostructure formation and ripple-like structure appearance on the surface, which is caused by the movement of polymer segments near *T*_g_. The ripple-like structure formation was previously observed also for gold [3] and plasma-ablated PLLA [12]. The effect of ripple-like structure formation is connected with a significant increase of surface roughness, directly proportional to the thickness of the prepared Pt layer. The width of ripples increases with Pt thickness as in the case of Au [3]. The ripple-like structure is more pronounced for the smaller thicknesses, and the worm-like structure appears for the Pt thickness of 50 nm when the PLLA is heated (Figure 3). The ripple-like formation can be possibly explained by the different thermal conductivities of Pt and PLLA, which induce changes in the PLLA system when heated at 60°C and cooled at room temperature. The depth diffusion of Pt (influencing the particle density) may be also an important factor for the ripple-like formation (see next paragraph).

**Figure 4:**
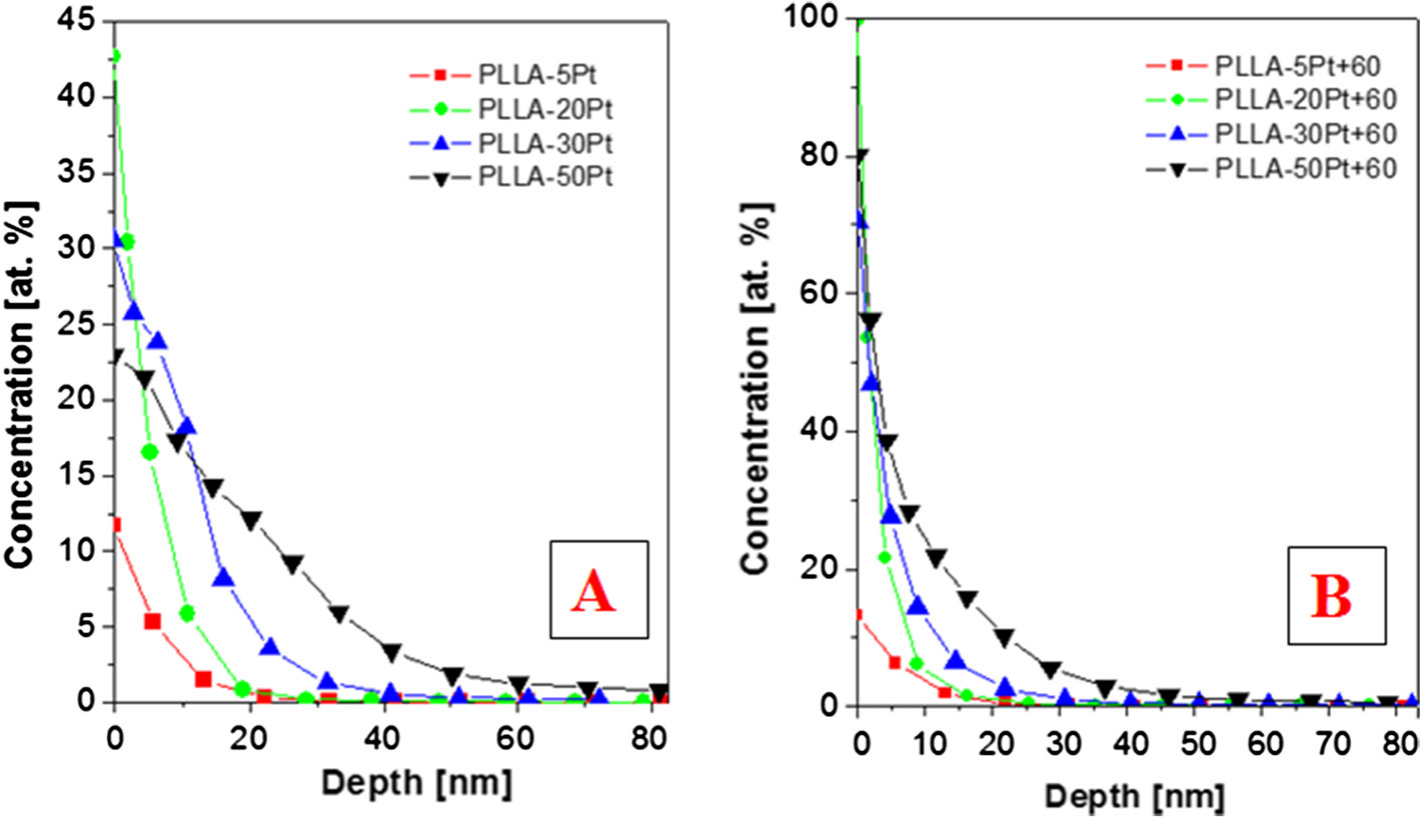
**Depth concentration profile of Pt for non-annealed and annealed samples measured by RBS.** (**A**) Non-annealed PLLA samples deposited with Pt (5, 20, 30 and 50 nm). (**B**) PLLA samples subsequently annealed at 60°C.

The depth concentration profile of platinum for non-annealed samples (Figure 4A) and thermally annealed samples (Figure 4B) was studied by RBS. From Figure 4, it can be concluded that in the sputtering process, slight diffusion of Pt atoms into the PLLA layer’s surface occurs. It is surprising that the surface concentration of Pt (except for the smallest thickness) decreases with an increasing Pt thickness. The reason for that might be the diffusion of Pt atoms into the polymer bulk. After annealing, the samples exhibited significantly higher values of Pt concentration on the very surface in comparison with non-annealed ones. The dramatic increase of surface roughness is exhibited in the annealed samples in comparison with the non-annealed samples (see Figure 3), e.g. *R*_a_ for the sample with 50 nm of Pt, the roughness increases *ca.* four times due to thermal annealing. The increase of Pt concentration on top of the ‘ripple-like’ structures is probably caused by the crystallization process of the PLLA substrate in combination with backward diffusion of Pt nanoparticles. During the crystallization process, local nucleation may play a significant role, being influenced by the amount of PLLA crystalline phase. Diffusion of Pt nanoparticles towards the top of the ripple structure is influenced by the different amounts of Pt incorporated in amorphous and crystalline phases. The inhomogeneous temperature and solidification of the PLLA surface is causing consequent Pt diffusion. The Pt concentration in the PLLA-Pt composite after annealing lies within the backward diffusion of Pt at higher temperature due to possible rotation and movement of short polymer segments near *T*_g_. These changes in Pt concentration may have also a significant role in ripple-like structure formation.

**Figure 5:**
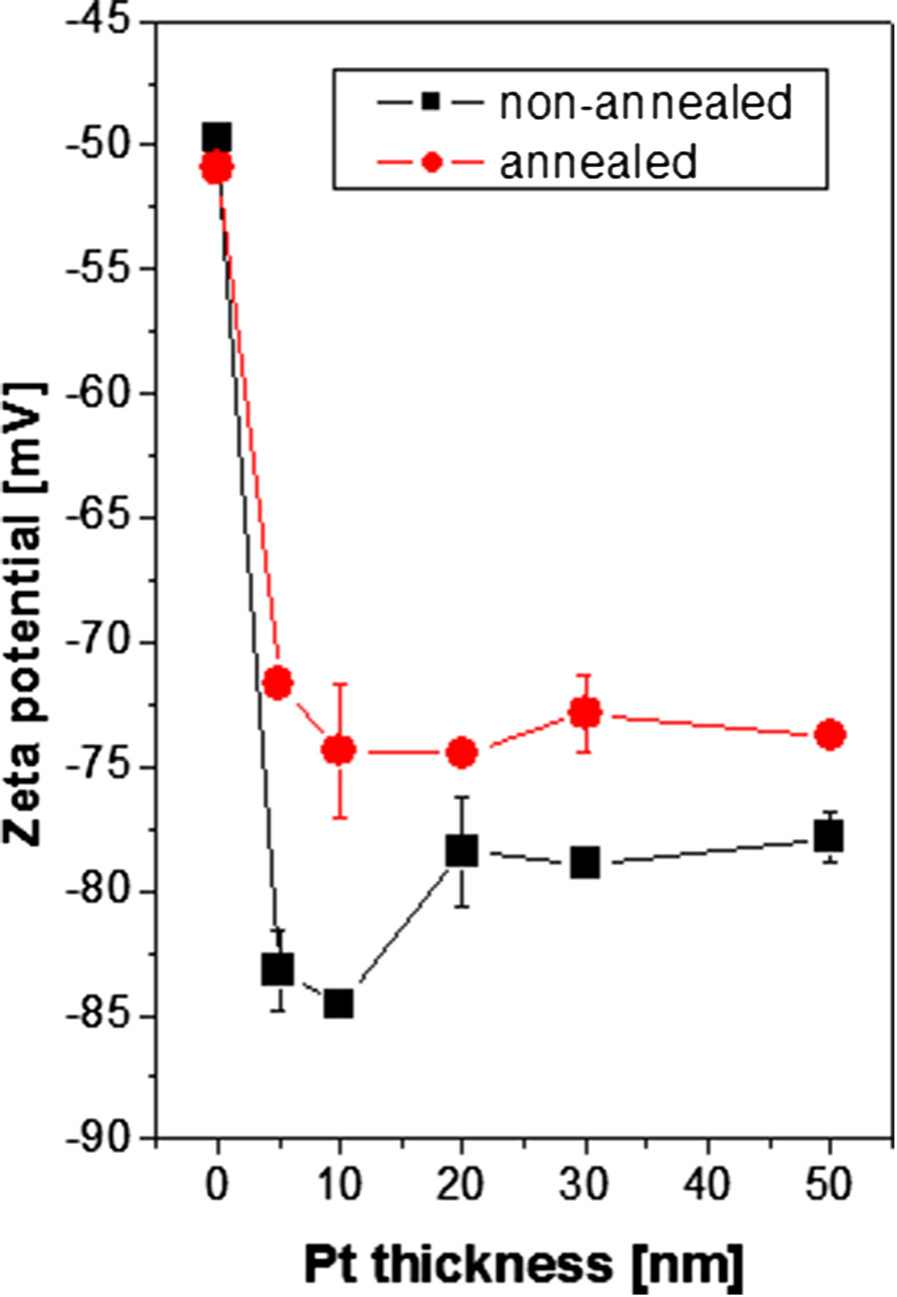
**Dependence of zeta potential on thickness of Pt sputtered layer for non-annealed and annealed samples.** As determined by streaming current method and the Helmholtz-Smoluchowski equation.

The zeta potential of studied samples is presented in Figure 5. It is obvious in the non-annealed samples (Figure 5, black) that after sputtering of Pt, the zeta potential decreases due to the presence of metal structures on the polymer surface and it does not change with increasing sputtering time (thickness) with respect to experimental error (of 10%). It indicates that the surface is totally covered by metal nanolayers after the shortest sputtering time (thinnest Pt layer). Also, the annealed samples (Figure 5, red) have the same trend. While in the non-annealed samples the zeta potential indicating total coverage is at 5 nm, in the annealed ones, it is at 10 nm. It corresponds well with sheet resistance measurement (see Figure 1). For thinner Pt layers, zeta potential values of annealed samples are quite higher due to increasing surface roughness and morphology and a partially uncovered polymer surface (see Figure 3) in comparison with those of non-annealed samples. However, for thicker Pt layers, zeta potential values of non-annealed and annealed samples are almost the same. This behaviour of Pt nanostructures is slightly different from that of Au nanostructures on polytetrafluoroethylene (PTFE) [24] and completely different from that of sputtered Ag nanostructures [25]. In the case of Au, the zeta potential decreased due to the creation of gold nanostructures, which take a part in the formation of negative surface charge on the contact with electrolyte due to accumulation of electrons in the case of metal-solution interface [24]. For the annealed samples, the dependence on the layer thickness is quite different. The annealing of Au nanostructures leads to a significant increase of the zeta potential for very thin layers due to thermal degradation (300°C) of PTFE accompanied by the production of excessive polar groups on the polymer surface, which plays an important role when the gold coverage is discontinuous [24]. The behaviour of the non-annealed Ag nanostructures is completely different, showing higher zeta potential in comparison with that of pristine PTFE explained by the creation of positive surface charge. Consequently, Ag sputtering (in Ar plasma) causes the formation of Ag^+^/Ag_2_O on the surface [25]. In the contact with a water-based electrolyte, Ag_2_O is hydrated into AgOH, which dissociates into Ag^+^ and OH^−^ ions, and the surface is positively charged. It is assumed that the Ag/polymer annealing results in Ag aggregation and, in turn, in partial uncovering of the PTFE surface and the zeta potential is probably first affected by the substrate surface.

## Conclusions

We have developed a simple and cheap procedure for biopolymer surface nanostructuring. The ripple patterns with a defined chemical structure, dimension and roughness were prepared. The platinum nanolayer sputtered on pristine PLLA surface becomes electrically continuous for a thickness of 5 nm. Thermal annealing results to the shift of the point of electrical continuity up to 10 nm. The annealing of the Pt-deposited polymer surface results in the formation of a ripple-like nanostructure. The surface roughness of the composite structure and its properties strongly depend on the thickness of previously sputtered Pt. The annealing of PLLA-Pt samples leads to a significant increase of metal concentration in the upper polymer layer and plays a significant role in ripple-like structure formation. Electrokinetic analysis confirmed the slightly different behaviour of as-sputtered and annealed Pt nanostructures, the highest differences being observed for lower Pt thicknesses.

## Competing interests

The authors declare that they have no competing interests.

## Authors’ contributions

PS analyzed the surface morphology, evaluated the surface roughness and thickness and designed the study. PJ carried out the sample preparation and participated on its analysis. ZK analyzed the zeta potential of the as-sputtered and annealed samples. PM and AM performed the RBS analysis. IM participated on the AFM analysis and proof corrections. VŠ participated in the study coordination and paper correction. All authors read and approved the final manuscript.
